# Matrix metalloproteinase 12 is an independent prognostic factor predicting postoperative relapse of conventional renal cell carcinoma - a short report

**DOI:** 10.1007/s13402-021-00650-9

**Published:** 2021-12-11

**Authors:** Bence Beres, Maria Yusenko, Lehel Peterfi, Gyula Kovacs, Daniel Banyai

**Affiliations:** 1 Department of Urology, Medical School, University of Pecs, Pecs, Hungary; 2 Institute of Biochemistry, University of Muenster, Muenster, Germany; 3 Medical Faculty, Ruprecht-Karls-University, Heidelberg, Germany

**Keywords:** Conventional renal cell carcinoma, Sarcomatous renal cell carcinoma, MMP12, Immunohistochemistry, Prognosis

## Abstract

**Purpose:**

Approximately 15% of clinically localised conventional renal cell carcinomas (cRCC) develop metastases within 5 years of follow-up. Sarcomatous cRCC is a highly malignant cancer of the kidney. The aim of our study was to identify biomarkers for estimating the postoperative progression of cRCCs.

**Methods:**

Global microarray-based gene expression analysis of RCCs with and without sarcomatous changes revealed that a high MMP12 expression was associated with a sarcomatous histology. Additionally, we analysed MMP12 expression using a multi-tissue array comprising 736 cRCC patients without metastasis at the time of surgery. The median follow-up time was 66 ± 29 months.

**Results:**

Immunohistochemistry revealed MMP12 expression in 187 of 736 cRCCs with good follow-up data. Subsequent Kaplan–Meier analysis revealed that patients with MMP12 positive tumours exhibited a significantly shorter tumour-free survival (*p* < 0.001). In multivariate Cox regression analysis a weak to strong MMP12 expression indicated a 2.4–2.8 times higher risk of postoperative tumour relapse (*p* < 0.001; *p* < 0.003, respectively).

**Conclusions:**

MMP12 may serve as a biomarker to estimate postoperative cRCC relapse and as a possible target for penfluridol therapy.

## Introduction

Conventional RCCs (cRCCs) make up 85% of all renal malignancies [1]. Approximately 20–25% of patients diagnosed with cRCC already carry metastases at the time of presentation. Metastatic cRCCs are resistant to chemo- and radiotherapy and show low responses to targeted therapies [2]. Currently, early diagnosis in conjunction with surgery is the best option to treat cRCC, whereas adjuvant therapy can only prolong the life of patients with metastatic disease.

As a result of widespread use of imaging techniques, a growing number of patients is diagnosed with incidentally detected small renal masses confined to the kidney [3]. The number of incidentally detected pT1a (< 4 cm in diameter) and pT1b (< 7 cm in diameter) tumours is increasing in the operation statistics of most urological centres. However, approximately 15% of clinically localised cRCCs operated with curative intent will develop metastases within 5 years. In the case of pT1 cRCC confined to the kidney, TNM classification cannot be used to estimate the postoperative course.

It is generally accepted that cRCC arises from proximal tubules of the adult kidney. During development and progression, the vast majority of cRCCs retain their epithelial characteristics. However, the most aggressive variants of cRCC undergo epithelial-mesenchymal transition (EMT) by gradually loosing epithelial characteristics and gaining a sarcomatous histology [4]. During EMT tumour cells loose the expression of several membrane proteins that play a seminal role in the maintenance and function of normal polarised proximal tubular cells. The invasive and metastatic growth of cRCC relies not only on the gain of a fibroblast-like/rhabdoid histology, but also on the capacity to degrade the basement membrane and modify the extracellular matrix [5].

Here, we analysed global gene expression patterns of cRCCs and papillary RCCs (pRCC) and of those exhibiting a sarcomatous histology and a rapid progression. We identified MMP12 as the most significantly overexpressed gene in sarcomatous cRCCs. Subsequent immunohistochemical analysis of a large cRCC cohort revealed that the expression of MMP12 significantly correlates with postoperative cRCC relapse confined to the kidney at the time of surgery.

## Materials and methods

### Microarray-based gene expression analysis

RCC samples and corresponding normal kidney samples were collected at the Department of Urology, University of Heidelberg, Germany in the period 1995–1996. Homogeneous areas of the tumour specimens were snap-frozen in liquid nitrogen immediately after operation and stored at –80 °C for subsequent analysis. In parallel, tumour specimens were fixed in 4% formaldehyde for histological examination. For global gene expression analysis we selected 17 cRCCs, 18 pRCCs with epithelial histology. as well as 3 cRCCs and 2 pRCCs with sarcomatous histology. The diagnosis of the tumours was confirmed genetically before use in this study. RNA was isolated using a Qiagen RNeasy Mini Kit (Qiagen, Hilden, Germany). Subsequent cDNA synthesis and hybridisation were performed at the Genomics Core Facility of the EMBL, Heidelberg using an Affymetrix Human Genome U133 Plus 2.0 array (Affymetrix, Santa Clara, CA, USA) containing 54.675 probes. Normalisation was performed using the R algorithm provided by Bioconductor. Differentially expressed genes were identified using Gene Set Enrichment Analysis (GSEA, www.broad.mit.edu/gsea). Visualisation of differentially expressed genes was performed using Multiple Array Viewer software (http://www.tm4.org/index.html). The expression profile data are deposited in the NCBI Gene Expression Omnibus under accession number GSEA 11151**.**

### Patients and tumour samples

The cohort used consisted of 736 patients subjected to radical or partial tumour nephrectomy between 2000 and 2015. The histological diagnosis and TNM classification was determined by one of the authors (GK) according to the Heidelberg classification and TNM systems applying 3 trier grading [6, 7]. We restrained to the Heidelberg Classification because it is based on robust tumour-specific genetic alterations and not on variable cytological characteristics. Approximately 70% of the cRCCs were composed of “clear” cells and the rest of “eosinophilic” (earlier called “granular”) cells, or mixed clear and eosinophilic cells. Data on regular follow-up and tumour-specific death were obtained from the Registry of the Department of Urology. Follow-up was defined as the time from operation until the last recorded control or cancer-specific death. Patients who died from causes other than RCC were not included in this analysis. Preoperative clinical staging included abdominal and chest computed tomography (CT) scans. Bone scans and brain CT scans were obtained only when indicated by clinical signs. The presence of nodal metastasis was confirmed by histological examination and that of distant metastases by radiographic examination. Postoperative patients were examined every 6 months by abdominal ultrasound and measurement of serum creatinine and eGFR, and every 12 months by CT scans.

### Tissue microarray (TMA) construction

Representative tumour areas were identified using haematoxylin and eosin stained slides and selected for TMA construction. From tumours with areas of different morphology or grading 2–4 biopsies were taken. Biopsies with a diameter of 0.6 mm were placed in a recipient block using a Manual Tissue Arrayer (MTA1, Beecher Instruments, Inc., Sun Prairie, USA). Foetal and adult kidney, brain and liver biopsies were included in the TMA.

### Immunohistochemistry

**Fig. 1:**
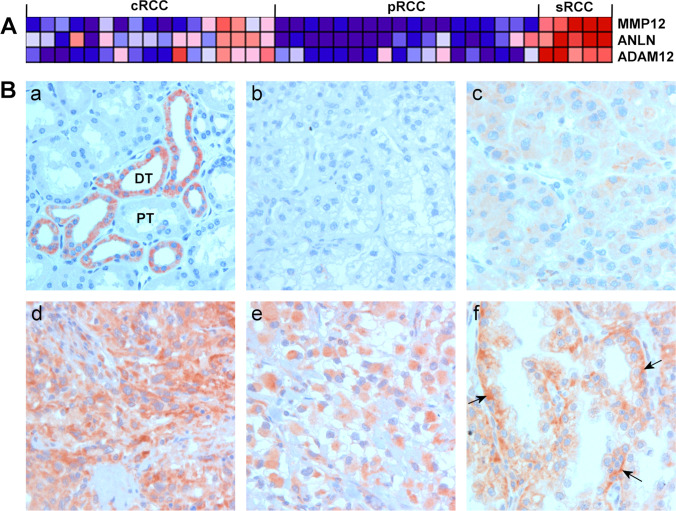
MMP12 expression in normal kidney and renal cell carcinoma (RCC). (**A**) Part of a heat map showing differential gene expression in conventional RCC (cRCC), papillary RCC (pRCC) and sarcomatous RCC (sRCC). The expression of MMP12, as well as that of ANLN and ADAM12, is upregulated in sRCC (red). None of the pRCCs, but 4 cRCCs without sarcomatous histology, exhibited a high MMP12 expression. (**B**) Immunohistochemistry of MMP12 expression. (a) Strong MMP12 expression in distal tubules (DT) of the adult kidney; proximal tubules (PT) are negative. (b) Lack of MMP12 expression in a cRCC. (c) Diffuse, weak MMP12expression in a cRCC with epithelial histology. (d) Strong MMP12 expression in a sRCC. (e) Strong cytoplasmic MMP12 expression in a rhabdoid cRCC. (f) Papillary growing epithelial cRCC displaying strong MMP12 expression in the basal regions of the tumour cells (arrows)

4 µm TMA sections were dewaxed in xylene and rehydrated in graded ethanol. Next, antigen retrieval was performed by boiling the slides in EnVision FLEX Target Retrieval Solution, high pH (DAKO, Glostrup, Danemark) in 2100-Retriever (Pick-Cell Laboratories, Amsterdam, The Netherlands). Endogenous peroxidase activity and nonspecific staining were blocked using an Envision FLEX Peroxydase Blocking Reagent (DAKO) for 10 min at room temperature. The resulting slides were subsequently incubated for one hour in a moist chamber with an anti-MMP12 antibody (NBP1-31225, Novus Biologicals*,* Littleton, CO, USA) at 1:250 dilution. EnVision, followed by a FLEX horse-radish-peroxydase conjugated secondary antibody (DAKO) for 30 min at room temperature. As a negative control, slides were incubated with only the secondary antibody. The signals were visualized with DAB (3,3’-Diaminobenzidin) (DAKO). Tissue sections were counterstained with Mayer's haematoxylin (Lillie’s modification, DAKO) and, after 10 s bluing in ammonium-hydroxide solution, mounted in Glycergel (DAKO). The immune reactions were evaluated by BB and GK blinded to the clinical data. Photographs were taken using a Leitz DMRBE microscope, equipped with a HC PLAN APO 20 × 0.70 objective, and a ProgRes C14 camera. Since the percentage of positively stained cells represented at least 90% of tumour cells in all positive biopsies, we did not evaluate the number of positive cells as a parameter. We classified the staining intensity as no staining, weak staining or strong staining (see Fig. 1 b-d).

### Statistical analysis

Data analysis was performed using SPSS Statistics software package version 20.0 (IBM,35 Armonk, NY, USA). Correlations between MMP12 expression and clinical pathological parameters were assessed using the Chi-square test. The effect of the different variables (age, sex, size of tumour, TNM classification, grade, stage and expression of MMP12) on the survival time of the patients was estimated using Kaplan–Meier analysis. Comparisons of survival curves were made using the Log rank test. Univariate and multivariate survival analyses were performed using the COX regression model. Patients alive and disease-free were censored. Differences were considered significant at *p* < 0.05.

## Results and discussion

We evaluated the expression profiles of 17 cRCCs and 18 pRCCs with epithelial histology against those of three cRCCs and two pRCCs with sarcomatous histology. Next, we selected 50 genes by Gene Set Enrichment Analysis (GSEA) that were upregulated in the sarcomatous RCCs. The results of the three most prominently expressed genes (MMP12, ANLN and ADAM12) are shown in Fig. 1A. MMP12 was overexpressed in four aggressively growing epithelial cRCCs as well. None of the pRCCs exhibited MMP12 overexpression.

MP12 expression was detected exclusively in distal tubular cells of normal foetal and adult kidneys, whereas proximal tubular cells were negative (Fig. 1B, a). Immunohistochemistry revealed a weak or strong cytoplasmic MMP12 staining in 187 of the 736 cRCCs, whereas 549 cRCCs were negative (Fig. 1B, b-f). The positive staining was restricted to the tumour cells, and no MMP12 protein was detected in the tumour microenvironment with the exception of some tumour-associated macrophages. Most of the weak positive cRCCs exhibited an epithelial histology, whereas tumours with a strong MMP12 expression exhibited sarcomatous or rhabdoid characteristics (Fig. 1B. d, e). In several tumours an accumulation of MMP12 protein was seen at the tumour-stroma border (Fig. 1B f).

Of the 736 cRCC patients, 426 (58%) were male and 310 (42%) were female The mean age of the patients was 60.9 ± 11.2 years (range 23–88 years). The average tumour size was 49.5 ± 25.3 mm. During a median follow-up of 66 ± 29 months, tumour relapse was observed in 119 patients (16%). Of the 736 tumours 574 (78%) were classified as pT1. The majority of the cRCCs (510 of 736; 69%) exhibited tumour grade G1. Regarding tumour stage, 668 (91%) of the cases were classified as stage I or II. Associations between MMP12 expression and clinical-pathological parameters such as postoperative tumour relapse and size, grade, T-stadium and tumour stage, as well as coagulation necrosis are depicted in Table 1. All parameters showed a significant correlation (*p* < 0.001) with MMP12 expression.

**Table 1:** Association of MMP12 expression with clinical-pathological parameters of conventional RCCs without metastasis at the time of operation (*n* = 736)

		Nr of cases (736)	MMP12 expression			*p*-value
			Negative (549)	Weak (128)	Strong (59)	
Gender						0.078
	Male	426	308	76	42	
	Female	310	241	52	17	
Status						< 0.001
	AWD	617	508	86	23	
	PTR	119	41	42	36	
Size						< 0.001
	< 4 cm	301	246	50	5	
	4-7 cm	286	215	42	29	
	> 7 cm	149	88	36	25	
T Stadium						< 0.001
	pT1	574	463	85	26	
	pT2	99	69	20	10	
	pT3	63	17	23	23	
Necrosis	No	658	517	103	39	< 0.001
	Yes	78	32	25	20	
Grade						< 0.001
	G1	510	436	60	14	
	G2	177	99	54	24	
	G3	49	14	14	21	
Stage						< 0.001
	I	570	461	84	25	
	II	98	68	20	10	
	III	68	20	24	24	

*AWD* alive without disease, *PTR* postoperative tumour relapse

**Fig. 2:**
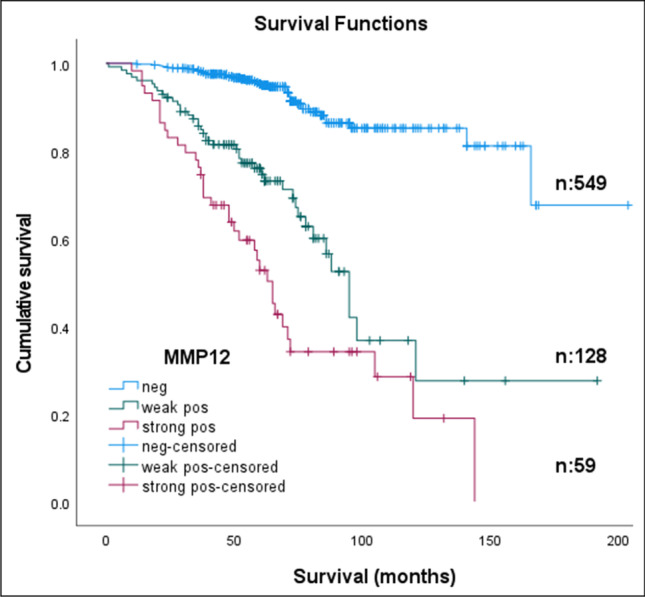
Kaplan–Meier estimates of recurrence-free survival according to immunohistochemistry in 736 patients without metastatic disease at the time of surgery. Weak or strong MMP12 expression reflects its prognostic value (*p* < 0.001)

Kaplan–Meier analysis revealed that cRCC patients exhibiting a weak or strong MMP12 expression had a significantly shorter disease-free survival compared to those without MMP12 expression (Fig. 2). The 5-year overall survival rates for the MMP12 strong, weak positive and negative groups were 52.8%, 75.2% and 95.3%, respectively. The mean survival for patients with a strong MMP12 staining was 74 (61–87) ± 7 months, with a weak staining 105 (85–125) ± 10 months and with a negative staining 176 (164–188) ± 6 months, with an overall survival of 154 (143–164) ± 5 months. Univariate Cox regression analysis revealed that tumor size, grade, T classification, necrosis and MMP12 positivity were significantly associated with postoperative tumour progression (all *p* < 0.001). However, in multivariate Cox regression analysis only tumour grade, stage and MMP12 positivity remained as independent predictors of relapse. In this correlation a weak or strong MMP12 staining indicated a 2.4–2.8 times higher risk of postoperative tumour relapse (*p* < 0.001 and *p* < 0.003, respectively) (Table 2).

**Table 2:** Multivariate analysis: expression of MMP12 protein is an independent prognostic factor indicating 2–3 times higher risk of cancer relapse (*p* ≤ 0.001; *p* ≤ 0.003)

	RR	95.0% CI for Exp(B)		*p*-value
		Lower	Upper	
Sex	1.015	0.690	1.492	0.941
Size				0.022
Size < 7 cm	1.807	0.931	3.506	0.080
Size > 7 cm	0.954	0.408	2.229	0.913
T1				0.855
T2	1.160	0.163	8.240	0.882
T3	1.456	0.315	6.729	0.631
G1				0.033
G2	1.785	1.091	2.920	0.021
G3	2.181	1.151	4.133	0.017
Stage I				0.032
Stage II	1.767	1.081	2.888	0.023
Stage III	2.218	1.173	4.194	0.014
MMP12 negative				< 0.001
MMP12 weak positive	2.785	1.717	4.518	< 0.001
MMP12 strong positive	2.391	1.344	4.254	0.003

It is generally accepted that cRCC arises from proximal tubules of the kidney, which is of mesodermal origin. During kidney development blastemal cells undergo mesodermal to epithelial transition (MET) to form polarized cells of the proximal tubular system. In highly malignant sarcomatous cRCC the opposite biological process, i.e., EMT, occurs resulting not only in loss of the polarized epithelial character of the tumour cells, but also in loss of cellular contact [4]. Simultaneous changes in the tumour microenvironment (TME), including changes in the extracellular matrix (ECM), may pave the way for invasive growth and metastasis [8]. The ECM is composed of fibrillar and non-fibrillar proteins, soluble extracellular proteins, cytokines and ECM-degrading enzymes such as MMP12. During metastasis, tumour cells break through the basal membrane separating them from the TME, thereby invading the TME and, next, blood vessels. In this complex process tumour-associated fibroblasts and immune cells play fundamental roles by producing interleukins, growth factors and matrix degrading enzymes [9]. Tumour cells can also modify their own microenvironment by producing growth factors and matrix degrading enzymes such as MMP12. As MMPs play an important role in the metastatic growth of tumours, they may be of prognostic importance [10]. MMPs are known to be involved in normal physiological processes such as embryonic development, wound healing and tissue remodelling, and to support the invasive growth and spreading of cancer cells [11, 12]. MMP12 expression has been found to be associated with the progression of several cancers [13–16]. In addition to its role in eliminating physical barriers, MMP12 can generate proangiogenic factors such as VEGF to form new blood vessels necessary for tumour growth [8]. Conversely, it has been found that MMP12 may have an anti-tumourigenic effect through hydrolysis of plasminogen to form the potent angiogenesis inhibitor angiostatin [17–19]. Therefore, MMP12 may have tumourigenic as well as anti-tumourigenic effects, depending on the type of tissue involved.

Here, we show that MMP12 acts as a pro-tumourigenic factor in cRCC. MMP12 expression correlates significantly with the occurrence of postoperative cRCC relapse. MMP12 expression may be employed for the identification of patients with a high risk of tumour progression, for close postoperative monitoring and for applying targeted therapy as early as possible. Recently, it has been shown that high MMP12 expression may be associated with the progression of lung adenocarcinoma and that penfluridol treatment may restrain the migration and metastatic growth of MMP12 expressing tumour cells [20]. We suggest that MMP12 may also serve as a therapeutic target for penfluridol in cRCC.

## Data Availability

Full data will be available from the corresponding author upon reasonable request.
